# DeBouganin Diabody Fusion Protein Overcomes Drug Resistance to ADCs Comprised of Anti-Microtubule Agents

**DOI:** 10.3390/molecules21121741

**Published:** 2016-12-17

**Authors:** Shilpa Chooniedass, Rachelle L. Dillon, Arjune Premsukh, Peter J. Hudson, Gregory P. Adams, Glen C. MacDonald, Jeannick Cizeau

**Affiliations:** 1 Viventia Bio Inc., Winnipeg, MB R3T 3Z1, Canada; RDillon@viventia.com (R.L.D.); APremsukh@Viventia.com (A.P.); JCizeau@viventia.com (J.C.); 2 Avipep Pty Ltd., Parkville 3052, Victoria, Australia; Peter.Hudson@avipep.com.au; 3 Eleven Biotherapeutics, Philadelphia, PA 19104, USA; GAdams@viventia.com; 4 Eleven Biotherapeutics, Winnipeg, MB R3E 0W3, Canada; glen.macdonald@eleven.com

**Keywords:** immunotoxin, deBouganin, ribosome inactivating protein, HER2, C6.5 diabody

## Abstract

Antibody drug conjugates (ADC), comprised of highly potent small molecule payloads chemically conjugated to a full-length antibody, represent a growing class of therapeutic agents. The targeting of cytotoxic payloads via the specificity and selectivity of the antibody has led to substantial clinical benefits. However, ADC potency can be altered by mechanisms of resistance such as overexpression of efflux pumps or anti-apoptotic proteins. DeBouganin is a de-immunized variant of bouganin, a ribosome-inactivating protein (RIP) that blocks protein synthesis, thereby leading to apoptosis. When conjugated to trastuzumab (T-deB), deBouganin was more potent than ado-trastuzumab-emtansine (T-DM1) and unaffected by resistance mechanisms to which DM1 is susceptible. To further highlight the differentiating mechanism of action of deBouganin, HCC1419 and BT-474 tumor cells that survived T-DM1 or trastuzumab-MMAE (T-MMAE) treatment were treated with an anti-HER2 C6.5 diabody–deBouganin fusion protein or T-deB. C6.5 diabody–deBouganin and T-deB were potent against HCC1419 and BT-474 cells that were resistant to T-DM1 or T-MMAE killing. The resistant phenotype involved MDR pumps, Bcl-2 family members, and the presence of additional unknown pathways. Overall, the data suggest that deBouganin is effective against tumor cell resistance mechanisms selected in response to ADCs composed of anti-microtubule payloads.

## 1. Introduction

Antibody Drug Conjugates (ADCs) are comprised of a full-length antibody chemically conjugated to small molecule payloads via a linker [1]. With the approval of brentuximab vedotin (SGN-35) and ado-trastuzumab-emtansine (T-DM1), along with more than 50 different ADCs currently in clinical trial, this class of anti-cancer agents represents a targeted drug alternative to the non-specific action of chemotherapeutic agents and radiation treatments [2,3,4]. The benefit of targeting highly potent payloads directly to the tumor cells using antibodies has resulted in a lower toxicity profile and meaningful clinical responses [5]. The potency of ADCs is generally linked to the antigen density, intracellular trafficking, and the release of the payload in the cytosol after lysosomal degradation of the antibody. Anti-microtubule agents such as mertansine (DM1) or monomethyl auristatin E (MMAE) and DNA binding agents such as calicheamicin, pyrrolobenzodiazepine dimer (PBD), or duocarmycin represent the majority of payloads currently in use for ADCs [6]. The average drug antibody ratio (DAR) varies between 2 and 4 and depends on the nature of the payload and the type of conjugation (random versus site-specific). However, due to their similarity to chemotherapeutic agents in term of size, chemical structure, and mechanism of action (MOA), the potency of these payloads can be affected by mechanisms of resistance such as multi-drug resistant (MDR) pumps or DNA repair mechanisms [7]. Moreover, other types of resistance specific to the antibody component of the ADC such as decreased levels of the cell surface target antigen, defective intracellular trafficking, and lysosomal degradation can reduce the amount of drugs reaching the cytosol [5,8,9,10]. Resistance to ADCs is seen in the clinic. In fact, despite favorable initial efficacy, the overwhelming majority of patients treated with T-DM1 eventually develop acquired resistance. In addition, infrequent cases of primary resistance are also reported [5]. These observations warrant the developments of payloads with distinct MOA that can overcome mechanisms of resistance affecting the efficacy of small molecule drugs.

Ribosome-inactivating proteins (RIPs) comprised of plant, fungi, algae, and bacterial toxins are *N*-glycosidase enzymes that deadenylate the 28S ribosomal RNA, thereby inhibiting protein synthesis and ultimately inducing cell death [11,12,13,14,15,16]. In addition, some RIPs also possess other mechanisms of cytotoxicity distinct from their capability to inhibit protein synthesis. These include the ability to induce DNA damage through the removal of adenines, the activation of the mitochondrial cell death pathway and the inhibition of the repair machinery that resolves DNA lesions generated by H_2_O_2_ [17,18,19,20]. The large family of RIPs from plants has been divided into two categories according to the number of polypeptide chains. Type 1 RIPs are monomeric proteins while type 2 RIPs are comprised of two subunits, an A-chain possessing RNA N-glycosidase activity and one lectin-like B-chain able to bind to cell surface polysaccharides [21]. The cytotoxicity of RIPs has generated interest about their applicability in oncology. As such, RIP-based immunotoxins are being evaluated as anti-cancer agents, using the specificity and selectivity of antibody fragments or scaffold proteins along with the potency of the toxin [22,23]. However, despite their potency, the immunogenicity of the toxin has limited the clinical use of immunotoxins, especially for the treatment of solid tumors. As a consequence, such drugs are suitable only for patients who have a compromised immune system or where the immunotoxin can be delivered via local administration such as squamous cell carcinoma of the head and neck and non-muscle invasive bladder cancer [24,25]. The clinical responses, observed in patients refractory to standard of care, have validated toxin proteins as clinically relevant payloads. This has warranted the development of a second-generation immunotoxin format allowing systemic administration, thereby permitting applications for a broader spectrum of indications.

Bouganin is a type 1 RIP isolated from *Bougainvillea* *spectabilis* Willd that demonstrates potent antitumor activity when delivered in the context of an antibody or antibody fragment [26,27,28]. A comparative study between bouganin and other RIPs including saporin and gelonin, chemically conjugated to anti-CD80 and anti-CD86 antibodies, showed that all conjugates killed in the pM range [29]. However, saporin conjugates were 1 to 2 logs more potent than the corresponding bouganin and gelonin conjugates. A de-immunized variant of bouganin, deBouganin, was created through the removal of T-cell epitopes, thus allowing repeat systemic administration and thereby addressing one of the major challenges facing immunotoxins. In an exploratory clinical trial, deBouganin genetically linked to an anti-EpCAM Fab fragment was well tolerated and demonstrated low immunogenicity as the majority of patients showed little to no antibody response to deBouganin [27]. A study comparing the biological activity of deBouganin conjugated to trastuzumab (T-deB) and T-DM1 highlighted deBouganin MOA versus the small molecule payload. Not only was a greater potency for deBouganin observed as compared to DM1 for the majority of high HER2 expressing cell lines. T-deB cytotoxicity was unaffected by a number of drug resistance mechanisms to which T-DM1 was susceptible, including MDR efflux pumps and modulation of apoptotic processes [30]. Furthermore, unlike small molecule payloads, a de-immunized protein toxin such as deBouganin offers the flexibility of being genetically linked to antibody fragments of varying sizes and formats or chemically conjugated to an IgG. Genetic linkage has several advantages including stable attachment of the toxin to the antibody fragment with a fixed DAR, thus precluding the need for site-specific conjugation strategies, the creation of homogeneous fusion proteins that are optimally sized for efficient tumor penetration, and economical bio-manufacturing.

In this report, we describe the engineering and biological activity of deBouganin genetically linked to an anti-HER2 C6.5 diabody, deB-C6.5-diab. DeB-C6.5-diab potency was similar to that of T-deB against a panel of breast cancer cell lines with different HER2 levels. Compared to clinically validated anti-microtubule agents, deB-C6.5-diab was more potent than T-DM1 and either more or equally as potent as T-MMAE against most HER2 3+ tumor cell lines. HCC1419 or BT-474 cells surviving a five-day exposure to T-DM1 or T-MMAE treatment were designated as HCC1419-T-DM1, HCC1419-T-MMAE, BT-474-T-DM1, or BT-474-T-MMAE, respectively. DeB-C6.5-diab was cytotoxic against these cell populations suggesting that deBouganin can overcome mechanisms of resistance developed against tubulin inhibitor agents. Overall, the potency of T-DM1 and T-MMAE against HCC1419 and BT-474 cells surviving T-DM1 or T-MMAE treatment was not restored in the presence of Bcl-2, Multidrug Resistance Associated Protein 1 (MRP1), P-glycoprotein or Multidrug Resistance Protein 1 (MDR1), and Breast Cancer Resistance Protein (BCRP) MDR pump inhibitors highlighting the multifaceted aspect of drug resistance to ADCs. Collectively, these results demonstrate that deBouganin’s distinct MOA could overcome mechanisms of resistance affecting the efficacy of anti-microtubule agents.

## 2. Results

### 2.1. Engineering and Selection of deB-C6.5 Diabody

**Figure 1:**
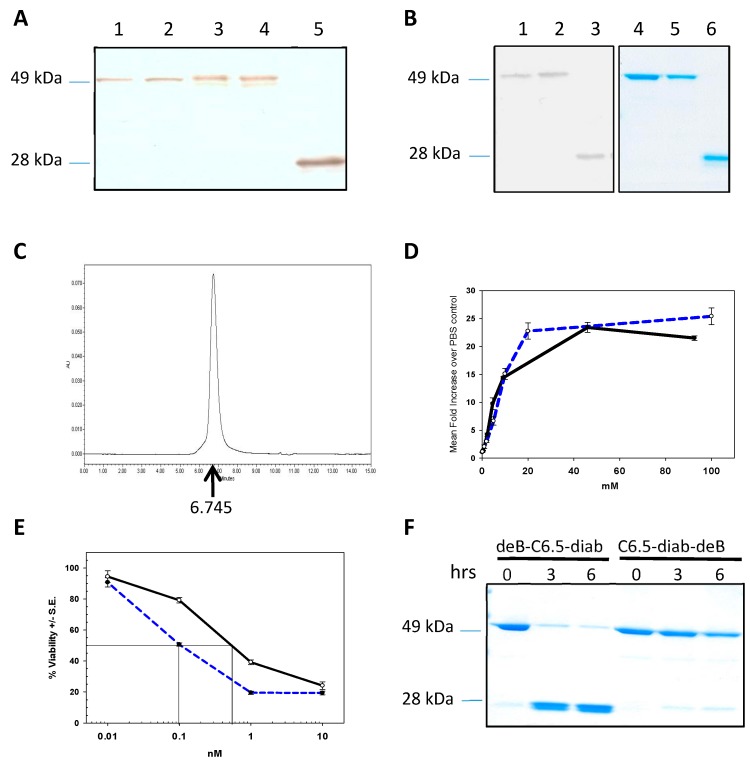
(**A**) Western Blot analysis of induced *E. coli* supernatants containing deB-C6.5-diab (lanes 1 and 2), C6.5-diab-deB (lanes 3 and 4), and C6.5-diab (lane 5) immunoblotted with an anti-His antibody; (**B**) Western Blot and Coomassie staining of purified deB-C6.5-diab (lanes 1 and 4), C6.5-diab-deB (lanes 2 and 5), and C6.5-diab (lanes 3 and 6). Purified samples resolved on an SDS-PAGE gel were either transferred to a nitrocellulose membrane and immunoblotted with an anti-His antibody (lanes 1, 2, and 3) or stained with Coomassie blue (lanes 4, 5, and 6); (**C**) SE-HPLC profile of purified deB-C6.5-diab with the retention time (6.745 min) indicated by the arrow; (**D**) Binding reactivity of C6.5-diab (dashed blue) and deB-C6.5-diab (black) against SK-BR-3 cells. Binding reactivity was determined by flow cytometry, as described in the Materials and Methods section; (**E**) Cytotoxicity of deB-C6.5-diab (dashed blue) and C6.5-diab-deB (black) against HER2-positive SK-BR-3 cells; (**F**) Kinetics of in vitro digestion of deB-C6.5-diab and C6.5-diab-deB by furin. Purified samples were incubated with recombinant furin enzyme for the indicated hours. Samples were then separated on an SDS-PAGE gel and stained with Coomassie Blue.

To create a deBouganin anti-HER2 recombinant protein, deBouganin was genetically linked to either the *N*- or *C*-terminus of the C6.5 diabody (V_H_-V_L_) via a peptidic linker containing the proteolytic furin site. The fusion constructs were designated as deB-C6.5-diab (deBouganin at the *N*-terminus of C6.5-diab) and C6.5-diab-deB (deBouganin at the *C*-terminus of C6.5-diab). After induction, the expression of soluble fusion proteins was assessed by Western blotting and compared to C6.5 diabody. As seen in Figure 1A, soluble deBouganin C6.5 diabody fusion proteins were found in the induced supernatant at the expected molecular weight.

Transformed *E. coli* clones were grown, induced, and the fusion proteins purified at a purity above 95%. After purification, a yield of 0.5 mg/L was obtained for deB-C6.5-diab and 0.14 mg/L for C6.5-diab-deB. To identify the construct with optimal biological activity, binding and potency of both fusion proteins were assessed. The titration curve of deB-C6.5-diab reactivity to HER2-positive cells was similar to that of equimolar concentrations of the control C6.5 diabody suggesting that the addition of deBouganin did not alter the diabody binding affinity (Figure 1B). The binding activity of purified deB-C6.5-diab and C6.5-diab was shown to be similar against the HER2-positive cell line SK-BR-3, while no binding was observed against the HER2-negative MCF-7 cell line, with either construct indicating the specificity of the diabody binding reactivity was unchanged (data not shown). The cytotoxicity of deB-C6.5-diab against SK-BR-3 was five times more potent than that measured for C6.5-diab-deB (Figure 1C). Of note, no IC_50_ was measured against the HER2-negative MCF-7 cell line with either of the deBouganin C6.5 diabody fusion proteins (data not shown). To possibly explain the difference in potency, both fusion proteins were incubated with purified furin protease and aliquots taken three and six hours post-incubation. As seen in Figure 1D, the proteolytic release of deBouganin from the diabody moiety was more efficient with the deB-C6.5-diab fusion protein. The more efficient release of protein payload from the diabody moiety, even if detected in non-physiological conditions, suggests that, upon internalization, a greater amount of deBouganin can reach the cytosol, thereby possibly explaining the increased potency of deB-C6.5-diab as compared to C6.5-diab-deB.

To assess whether the biological activity of deB-C6.5-diab could be further improved by changing the orientation of the variable domains, a deB-C6.5-diab V_L_-V_H_ variant was engineered. However, SE-HPLC analysis showed that the fusion protein was unstable and formed aggregates during purification. Therefore, based on the previous data, deB-C6.5-diab (V_H_-V_L_) was selected as the optimal anti-HER2 deBouganin diabody fusion protein format.

### 2.2. Biological Characterization of deB-C6.5-diab

**Figure 2:**
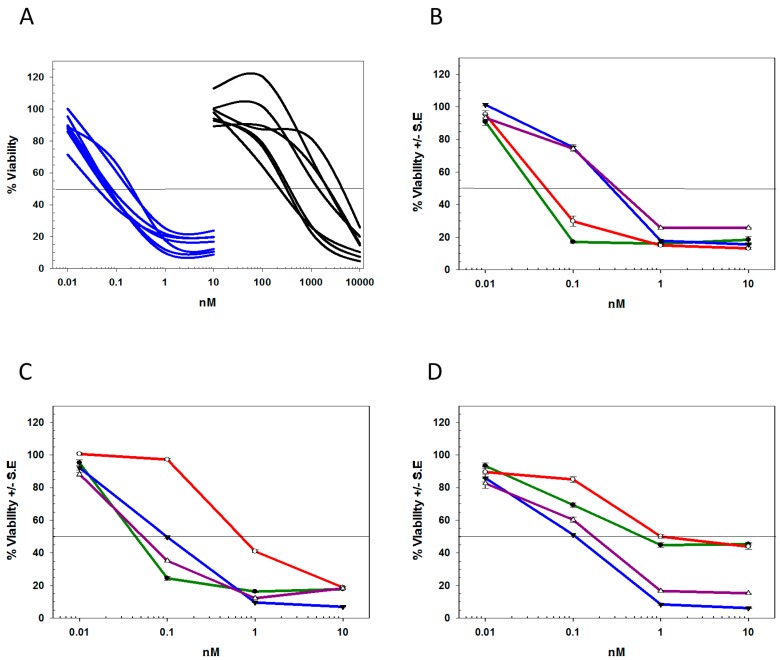
(**A**) Cytotoxicity of deB-C6.5-diab (blue) and deB (black) against a panel of HER2 3+ cell lines. Cytotoxicity of deB-C6.5-diab (blue), T-DM1 (red), T-MMAE (green); and T-deB (purple) against SK-BR-3 (**B**); BT-474 (**C**); and HCC1419 (**D**) cells.

To demonstrate the relationship between antigen density and cytotoxicity, deB-C6.5-diab was tested against a variety of cell lines expressing different levels of HER2. As seen in Table 1, double digit pM to subnanomolar IC_50_ values were measured for deB-C6.5-diab against most HER2 3+ and HER2 2+ tumor cells but did not show any significant cytotoxicity against HER2 1+ tumor cells. A comparison in IC_50_s against HER2 3+ tumor cells between free deBouganin and deB-C6.5-diab showed that, deB-C6.5-diab was, on average, at least 3 logs more potent than free deBouganin (Figure 2A).

The potency of deB-C6.5-diab was subsequently compared to that of trastuzumab conjugated with DM1 (T-DM1), MMAE (T-MMAE), or deBouganin (T-deB). The deB-C6.5-diab and T-deB IC_50_ values were comparable for all cell lines tested as both agents are bivalent and have a DAR of exactly (deB-C6.5-diab) or approximately (T-deB) two (Table 1) [30].

Similar to the previously published data [30], deB-C6.5-diab was more potent than T-DM1 against most HER2 3+ tumor cell lines except for SK-BR-3 cells. DeB-C6.5-diab and T-MMAE exhibited similar cytotoxicities against most HER2 3+ cell lines tested except for HCC1419 and HCC1569, where deB-C6.5-diab was a log more potent than T-MMAE. On the other hand, T-MMAE, like T-DM1, was more potent than deB-C6.5-diab against SK-BR-3 cells. The MTS curves of SK-BR-3, BT-474 and HCC1419 highlight the different killing efficiencies observed for the tested agents (Figure 2B–D). Against HCC1419, deB-C6.5-diab and T-deB exposure led to >80% cytotoxicity at 10 nM, whereas approximately 40% of the cells remained viable after T-DM1 or T-MMAE treatment. At 10 nM, the same level of cytotoxicity was achieved with all agents against BT-474 cells with the exception of T-DM1, which was a log less potent. Similarly, for SK-BR-3, only 20% of the cells remained viable after exposure to test agents at a 10 nM concentration; however, both deBouganin molecules were 10-fold less potent than either ADC.

**Figure 3:**
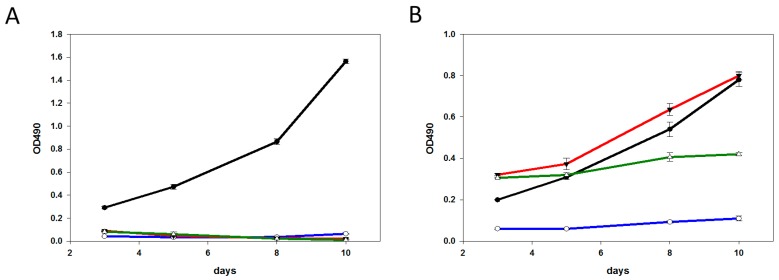

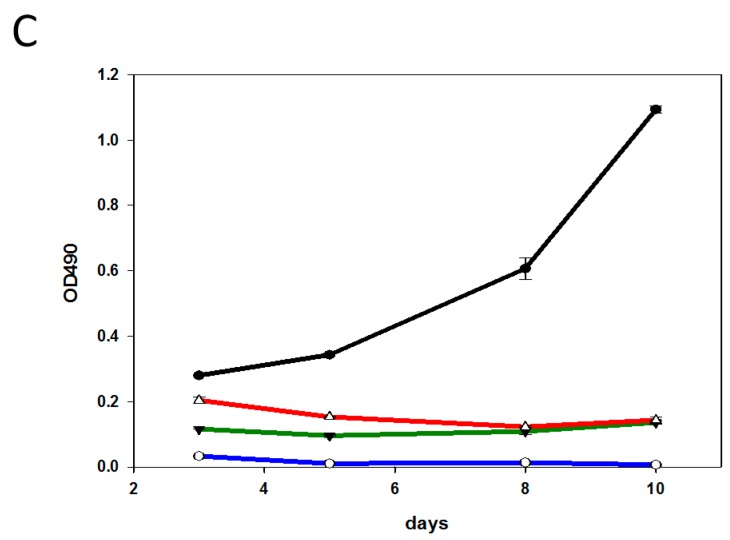
Growth profiles of SK-BR-3 (**A**); HCC1419 (**B**); or BT-474 (**C**) cells surviving deB-C6.5-diab (blue), T-DM1 (red), T-MMAE (green) treatments, or untreated cells (black). Untreated cells or cells surviving deB-C6.5-diab, T-DM1, and T-MMAE treatments were harvested and recultured without drugs. Cell growth was measured as O.D._490_ using the Cell Titer 96 Aqueous One solution cell proliferation assay at three, five, eight, and 10 days, as described in the Materials and Methods section.

To assess the growth characteristics of the HCC1419, BT-474, and SK-BR-3 cells surviving treatment, cells exposed to the different agents for five days were collected and cultured in adherent conditions. As seen in Figure 3, no significant growth was measured with deB-C6.5-diab-treated HCC1419, BT-474, and SK-BR-3 cells after three, five, eight, and 10 days, suggesting the remaining live tumor cells could not be rescued through re-culturing (Figure 3). Similar data was obtained with SK-BR-3 cells following treatment with T-DM1 or T-MMAE (Figure 3A). In contrast, the growth curve of HCC1419-T-DM1 cells was similar to that of untreated cells, whereas a slight decrease in proliferation was measured with HCC1419-T-MMAE cells (Figure 3B). The O.D._490_ of BT-474-T-DM1 or BT-474-T-MMAE cells did not change over time, indicating that cell replication had been impaired and observation of the well contents showed both dead and live cells (Figure 3C).

### 2.3. Differentiating deBouganin MOA from Anti-Microtubule Agents

**Figure 4:**
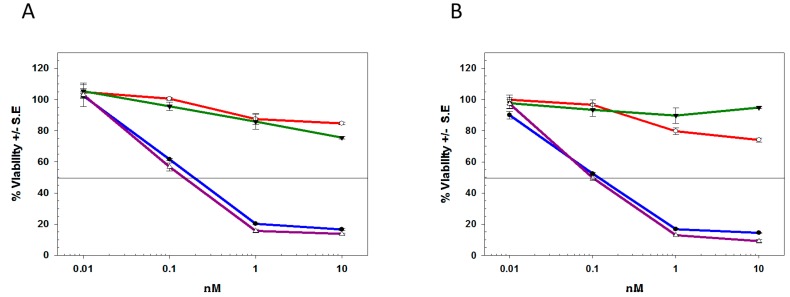
Cytotoxicity of deB-C6.5-diab (blue), T-DM1 (red), T-MMAE (green), and T-deB (purple) against HCC1419 cells surviving T-DM1 (**A**) or T-MMAE (**B**) treatments. Representative examples of two independent experiments.

To assess whether tumor cells surviving T-DM1 or T-MMAE killing were still sensitive to these cytotoxins, HCC1419-T-DM1, HCC1419-T-MMAE, BT-474-T-DM1, and BT-474-T-MMAE cells were re-challenged with T-DM1 or T-MMAE. As seen in Table 2 and Figure 4, BT-474 or HCC1419 cells that survived previous exposure to T-DM1 or T-MMAE were more resistant to any further killing by T-DM1 or T-MMAE with only approximately 20% decreased viability at 10 nM. Similar data were obtained with secondary exposure to taxol, DM1, or MMAE, suggesting that resistance to anti-microtubule agents was, at least in part, independent of antibody trafficking (Table 2). To evaluate a small molecule payload with a cell-cycle independent MOA, HCC1419-T-DM1, HCC1419-T-MMAE, BT-474-T-DM1, and BT-474-T-MMAE cells were incubated with T-duo or duocarmycin. As seen in Table 2, these cells were a log less sensitive to either T-duo or duocarmycin compared to their untreated counterparts. In comparison, deB-C6.5-diab and T-deB had similar cytotoxicities against untreated or treated HCC1419 or BT-474 cells with all IC_50_ values remaining in the sub-nanomolar range (Figure 4). To expand this observation to another protein toxin payload, an anti-EpCAM scFv (scFv-ETA), genetically-linked to a truncated form of *Pseudomonas* exotoxin A (ETA) which prevents protein synthesis via the ADP ribosylation of the elongation factor-2, was tested. scFv-ETA was also cytotoxic against T-MMAE or T-DM1 treated cells, demonstrating that protein payloads can reverse the resistance acquired against anti-microtubule agents (data not shown).

**Figure 5:**
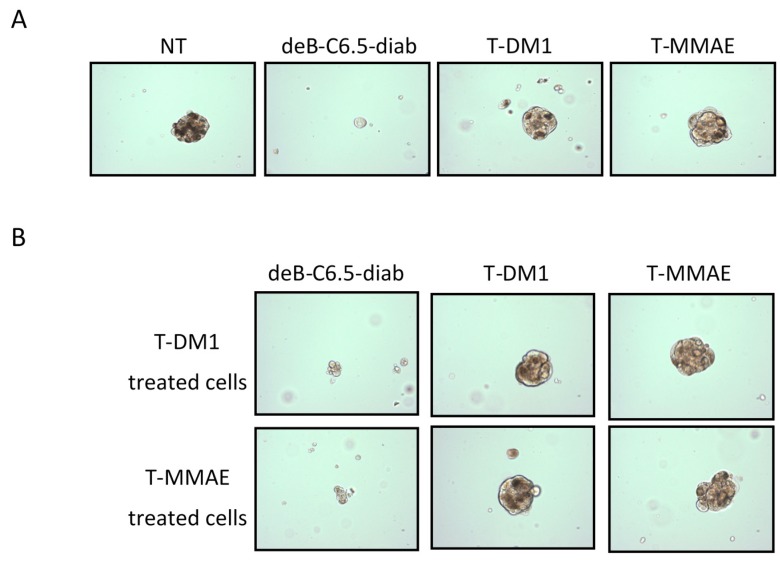
(**A**) Representative images of tumorospheres from untreated (NT) HCC1419 cells or cells treated with 10 nM deB-C6.5-diab, T-DM1, or T-MMAE. (**B**) Representative images of tumorospheres from HCC1419 cells surviving T-DM1 or T-MMAE treatment incubated under tumorosphere-forming conditions in the presence of 10 nM deB-C6.5-diab, T-DM1, or T-MMAE.

To assess whether the resistance mechanism was associated with a particular cell phenotype related to stem cells, the surviving HCC1419 and BT-474 cells were cultured under non-adherent conditions to promote stem cell colony formation. The number of tumorospheres observed in HCC1419-T-DM1, HCC1419-T-MMAE, BT-474-T-DM1, and BT-474-T-MMAE cultures was similar to untreated cells (Table 3 and Figure 5), suggesting that the resistance of the surviving cells was unrelated to “stemness”. T-DM1 or T-MMAE treated cells showed >97% tumorosphere inhibition when incubated with 10 nM deB-C6.5-diab. T-DM1 or T-MMAE had only a partial effect at inhibiting sphere formation in these treated cells (Table 3 and Figure 5B).

### 2.4. Characterization of the T-DM1 and T-MMAE Surviving Cells

To confirm that HER2 was not downregulated, T-DM1 and T-MMAE treated cells were analyzed by flow cytometry and compared to untreated cells. No difference was measured between treated BT-474 and HCC1419 cells and their respective untreated controls, suggesting that the level of HER2 and epitope accessibility was not affected by the treatment (data not shown).

**Figure 6:**
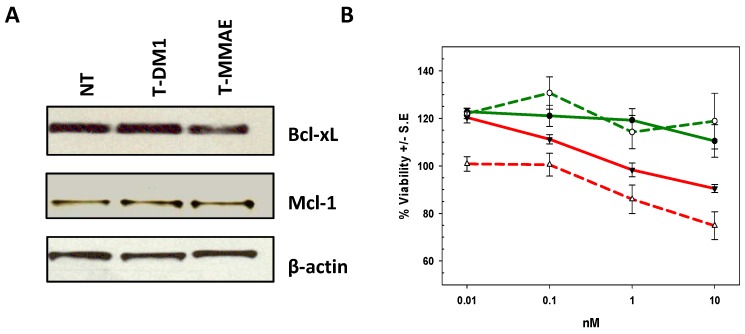
(**A**) Western blot analysis of the expression of Bcl-xL and Mcl-1 in untreated (NT) HCC1419 cells or cells surviving T-DM1 or T-MMAE treatment. β-actin levels were monitored to ensure equal loading; (**B**) Cytotoxicity of T-DM1 (red) or T-MMAE (green) in the presence (dashed lines) or absence (solid lines) of ABT-737 against HCC1419 cells that survived T-DM1 (red) or T-MMAE (green) treatment.

The identification of the pathways underlying T-DM1 or T-MMAE resistance would identify the mechanisms of resistance overcome by deBouganin and further highlight deBouganin differential MOA. Since earlier reports demonstrated that T-DM1 potency can be inhibited by the expression of Bcl-2 proteins, their potential upregulation within T-DM1 or T-MMAE treated cell populations was investigated by Western blot analysis. No significant upregulation of Bcl-xL or Mcl-1 levels in HCC1419-T-DM1 or HCC1419-T-MMAE versus untreated HCC1419 cells was seen (Figure 6A). Bcl-2 protein was not detected regardless of treatment or lack thereof (data not shown). However, treatment with ABT-737, a BH3 mimetic inhibitor of Bcl-2, Bcl-xL and Bcl-w marginally improved T-DM1 potency against HCC1419-T-DM1 cells, while no effect was seen against HCC1419-T-MMAE cells (Figure 6B).

**Figure 7:**
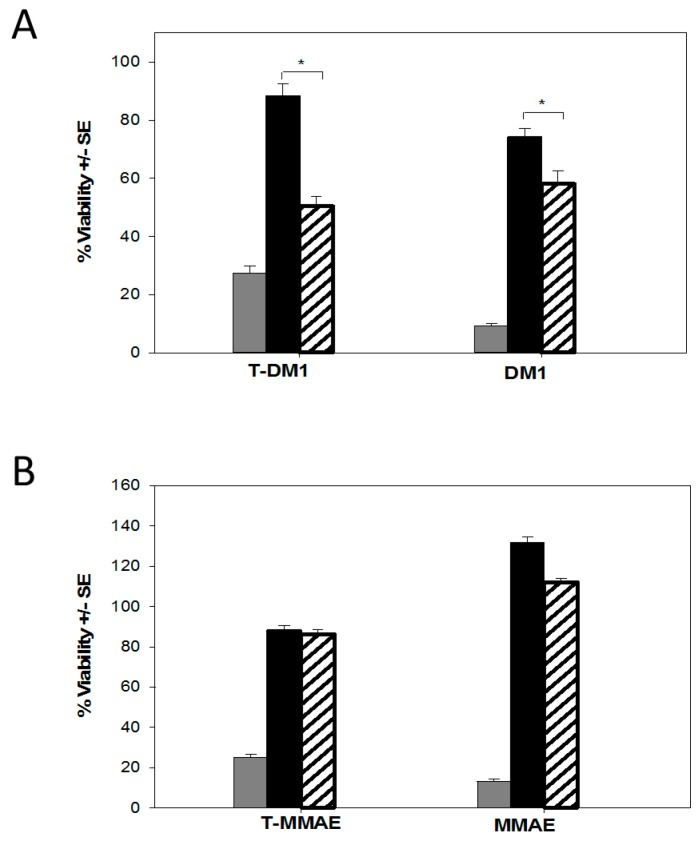
Role of BCRP in BT-474 cells that survived T-DM1 or T-MMAE treatments. (**A**) Cytotoxicity of T-DM1 and DM1 was measured against BT-474 cells (grey bars) and T-DM1 surviving BT-474 cells with (hashed bars) or without (black bars) 10 µM Ko143; (**B**) Cytotoxicity of T-MMAE and MMAE was measured against BT-474 (grey bars) and T-MMAE surviving BT-474 cells with (hashed bars) or without (black bars) 10 µM Ko143. * indicates significant difference (*p* < 0.05).

To assess if MDR pump upregulation was involved in T-DM1 and T-MMAE resistance, doxorubicin, a known MRP1 and P-glycoprotein substrate, and irinotecan, a known BCRP substrate, were tested against HCC1419-T-DM1 and HCC1419-T-MMAE cells. Both HCC1419 and BT-474 cells surviving T-DM1 or T-MMAE treatments were less sensitive to doxorubicin and irinotecan than their untreated counterparts (data not shown). In addition, the potency of doxorubicin and irinotecan was restored to various degrees in the presence of PSC833, MK-571, and Ko143, inhibitors of MRP1, P-glycoprotein, and BCRP, respectively (data not shown). Overall, this data confirmed that HCC1419 and BT-474 cells surviving T-DM1 or T-MMAE treatment express functionally active MRP1, MDR1, and BCRP pumps. To determine the involvement of each of these efflux pumps in T-DM1 and T-MMAE resistance, HCC1419-T-DM1 and HCC1419-T-MMAE cells were re-challenged with the respective ADC in the presence or absence of the MK-571, PSC833, and Ko143 agents. Interestingly, HCC1419-T-DM1, HCC1419-T-MMAE, and BT-474-T-MMAE cells did not show any increased sensitivity to either ADC in the presence of the MDR inhibitors (data not shown). The only effect was measured for BT-474-T-DM1 cells, which were partially re-sensitized to T-DM1 upon concomitant Ko143 treatment (Figure 7A). In addition, the sensitivity to DM1 or MMAE was also largely unaffected in the presence of inhibitor against resistant BT-474 and HCC1419 cells (data not shown). Only co-treatment with Ko143 partially sensitized BT474-T-DM1 cells to DM1 (Figure 7A). Since the data suggested that at least three different MDR proteins were present, the experiment was repeated in the presence of a combination of MK-571/PSC833 and MK-571/Ko143 inhibitors against HCC1419-T-DM1 and HCC1419-T-MMAE cells. However, the mixture of inhibitors did not lead to an increased potency of either ADC (data not shown). The lack of sensitization to T-DM1 or T-MMAE in the presence of the inhibitors tested suggests the presence of additional mechanisms of resistance that either encompass other efflux pumps or are distinct from MDR pumps. Nonetheless, deBouganin, whether conjugated to trastuzumab or genetically linked to the C6.5-diab, overcomes mechanisms of resistance that affect T-DM1 and T-MMAE potency.

## 3. Discussion

Trastuzumab therapy has markedly improved the poor prognosis associated with HER2-amplified breast cancer. However, most patients eventually experience disease progression [31,32]. Several other HER2-targeted agents have been evaluated with the hope of circumventing the resistance associated with trastuzumab. The ADC T-DM1 is one such agent that combines the targeting specificity of trastuzumab with the cytotoxic activity of the anti-microtubule agent DM1. While treatment with T-DM1 showed superior efficacy and a better safety profile as compared to therapy with trastuzumab alone or trastuzumab plus chemotherapy, the majority of these patients eventually cease to respond. Although incompletely understood, multiple factors have been implicated in T-DM1 resistance such as lower HER2 expression, epitope masking, tubulin alteration, defective intracellular trafficking, modulation of apoptotic or cell survival pathways, and drug efflux [5].

In this report, we identified HER2+ tumor cells lines with multiple mechanisms of anti-tubulin drug resistance. Evidence was presented that the resistance could be isolated to a subpopulation of cells following a single treatment. Surprisingly, this distinct cell population not only exhibited resistance on re-exposure but also demonstrated cross-resistance against other small molecule compounds with a similar and different MOA. In addition, treatment with T-DM1 or T-MMAE failed to prevent tumorosphere formation in untreated or resistant cells. Assessment of potential contributing mechanisms showed that some common MDR efflux pumps as well as Bcl-2 family members did play a role, but only in part, and that other MDR pumps or other as yet identified mechanisms may be involved. The identification of resistant mechanisms specific to each cell line showed that no one common pathway could be ascribed to the anti-tubulin resistance. As HCC1419 cells were derived from a cancer patient who had received chemotherapy [33], it is intriguing to speculate that the inherent resistance to anti-microtubule agents may have resulted during their anti-cancer treatment. If this is the case, the appearance of cross-resistance suggests that the choice of certain cytotoxic compounds in the design of ADCs may limit their efficacy when used as a follow-up treatment approach to chemotherapy.

In contrast to the anti-microtubulins, deBouganin, whether genetically linked to the anti-HER2 C6.5 diabody or chemically conjugated to trastuzumab, was cytotoxic against BT-474 and HCC1419 tumor cells irrespective of their drug resistance properties. Despite the moderate affinity of C6.5 relative to trastuzumab, deB-C6.5-diab potency was very similar to T-deB against HER2-positive tumor cell lines. The comparable potencies could in part be explained by the possible reduced intracellular delivery of deBouganin due to trastuzumab recycling to the cell surface [34]. In addition, the deB-C6.5-diab optimization for a faster and more efficient release of deBouganin likely resulted in a greater amount reaching the cytosol prior to degradation in the lysosome. Altogether, the cell surface recycling of T-deB and the optimized deBouganin release from the C6.5-diab moiety likely resulted in similar amounts of cytosolic deBouganin explaining the comparable IC_50_ values between both formats. Interestingly, no growth was measured after the re-culture of deB-C6.5-diab treated cells, demonstrating that deBouganin led to 100% cytotoxicity in vitro. Moreover, treatment with deB-C6.5-diab nearly eliminated tumorosphere formation in both cell lines, suggesting that the deBouganin payload was also cytotoxic against tumor cells possessing cancer stem cell-like properties.

The potency of anti-HER2 deBouganin immunotoxins against all HCC1419 and BT-474 cells regardless of prior treatment shows that the resistance to the anti-microtubulin agents was unlikely linked to decreased HER2 expression on the cell surface. Furthermore, the reduced potency observed with T-duo against treated BT-474 and HCC1419 cells ruled out the possibility that the resistance was linked to cell-cycle-dependent payloads or associated with tubulin mutations. Other mechanisms linked to the antibody such as altered intracellular trafficking and defective degradation of the ADCs in the lysosomes may also be involved. Therefore the results presented here highlight the heterogeneity of mechanisms within the BT-474- and HCC1419-resistant cells that may more closely resemble the clinical situation as opposed to cells derived from clonal selection.

T-DM1- or T-MMAE-resistant cells were also sensitive to an anti-EpCAM scFv genetically fused to a truncated form of ETA. This demonstrates that protein toxins can overcome the resistance properties of these cells in a manner independent of the targeting vehicle (i.e., fusion construct vs. full-length IgG), linker (peptidic versus chemically conjugated) and the cell surface target (i.e., HER2 vs. EpCAM). The potency of deBouganin and ETA also supports the notion that T-DM1 or T-MMAE treated cells will likely be sensitive to most protein payloads. The cytotoxicity of deBouganin, a 29 kDa protein, and other protein payloads versus cells expressing MDR pumps is not surprising due in part to their larger size compared to small molecules. Indeed, the potency of RIP proteins such as saporin, gelonin, and ricin or bacterial toxins such as ETA and diphtheria toxin has been reported against tumor cells overexpressing P-glycoprotein, BCRP, or different MRP1 family members [35,36,37]. This observation was translated clinically with an anti-CD22 scFv-ETA recombinant immunotoxin, BL22, in chemotherapy-resistant hairy-cell leukemia, where 11 out of 16 treated patients had complete remission. An affinity-matured version of the anti-CD22 immunotoxin, moxetumomab pasudotox, is currently being tested in a phase III study [38]. Similarly, in a phase I study, three out of 10 chemotherapy-refractory patients had durable partial responses with SSP1, an anti-mesothelin scFv-ETA. Moreover, two other refractory patients responded to chemotherapy after SSP1 treatment [39,40]. Interestingly, the possible deficient lysosome trafficking in resistant cells could potentially be advantageous for RIP toxins. Indeed, it is largely accepted that most type I RIPs like deBouganin escape the endocytic pathway via the endosomes before being degraded in the lysosome [16]. Therefore, deficient intracellular trafficking to the lysosome may allow a more efficient release of RIP protein from the endosomal compartment into the cytosol. Although not studied with deBouganin, it has been reported that RIPs can directly affect mitochondrial function. For example, loss of mitochondrial membrane potential and ROS production was observed with HL-60 cells and JAR cells treated with trichosanthin [41,42] and with MCF-7 cells treated with marmorin [43]. Other mechanisms such as an upregulation of the pro-apoptotic protein Bax along with a downregulation of the anti-apoptotic protein Bcl-2 was also observed with abrus agglutinin treated HepG2 cells. Therefore, we cannot exclude that other mechanism(s) of action distinct from inhibition of protein translation may account for the ability of deBouganin to overcome resistance induced by microtubule agents.

In summary, the data presented here suggest that treatment with ADCs comprised of anti-microtubule agents may not be effective against all tumor cells and can therefore lead to an outgrowth of a subpopulation with a broad drug-resistant phenotype. In contrast, deBouganin, a plant protein toxin designed for systemic delivery, was able to overcome the complex interplay of multiple mechanisms of resistance arising after treatment with anti-microtubule agents and therefore may represent a needed therapeutic approach for patients developing resistance to these agents.

## 4. Materials and Methods

### 4.1. Cell Culture

All tumor cell lines (American Type Culture Collection, Manassas, VA, USA) and OE-19 (Sigma, St. Louis, MO, USA) were cultured in their respective media as per the provider’s instructions in a humidified incubator at 37 °C in the presence of 5% carbon dioxide.

### 4.2. Antibodies, Toxins, and Conjugates

Mertansine (DM1), monomethyl auristatin E (MMAE), duocarmycin, T-MMAE (trastuzumab conjugated to MMAE via a valine-citrulline-p-aminobezoyloxycarbonyl (vc-PAB) linker), T-duo (trastuzumab conjugated to duocarmycin via a vc-PAB linker), and T-DM1 were obtained from Levena Biopharma (San Diego, CA, USA). T-deB was prepared by chemically conjugating trastuzumab and deBouganin as previously described [30].

### 4.3. Plasmid Construction

DeBouganin-C6.5 diabodies (in the V_H_-V_L_ and V_L_-V_H_ orientations) and C6.5-diab-deBouganin were engineered by the splice overlapping extension PCR method using deBouganin and C6.5 diabody DNA templates. The PCR fragments were cloned into the pCR 2.1 vector and sequenced. The pCR 2.1 plasmids containing the correct inserts were digested and ligated into the pING 3302 plasmid pre-digested with the corresponding restriction enzymes. In all constructs, the deBouganin and the C6.5 diabody moieties were genetically linked via a peptidic linker containing a proteolytic site for the furin protease (TRHRQPRGWEQL). All constructs were preceded by a 5′ PelB leader sequence from *Erwinia carotavora* and placed under the control of an arabinose inducible promoter. Recombinant immunotoxins were expressed as soluble proteins using E104 *E. coli* cells and secreted into the supernatant.

### 4.4. Research Scale Expression Study

Transformed E104 *E. coli* were propagated in Terrific Broth media (1% inoculum) to an O.D._600_ of 2. At this time, the culture was induced with 0.1% l-arabinose (+) (Sigma) and incubated at 25 °C overnight. After centrifugation, supernatants were analyzed by Western blot to confirm the presence and size of the fusion proteins. Briefly, induced supernatants were loaded on a SDS-PAGE gel and transferred to a nitrocellulose membrane. Diabody and diabody fusion proteins were detected with an anti-Histidine (His) antibody (GE Healthcare, Buckinghamshire, UK) at a 1/1000 dilution followed by a sheep anti-mouse IgG coupled to horseradish peroxidase (HRP) (Sigma) at a 1/1000 dilution. The signal was developed using the DAB reagent (Sigma).

### 4.5. Large-Scale Expression and Purification of Fusion Proteins

Fed batch fermentation was performed in a 20-L CHEMAP fermenter using glycerol minimum medium. The temperature was set at 28 °C and the pH maintained at 7.0 throughout the entire fermentation. At an O.D._600_ of 50, the culture was induced with an arabinose solution (50% glycerol and 30 g/l-arabinose solution) for 30 h. The culture supernatant was harvested by centrifugation at 8000 rpm for 30 min, followed by microfiltration and 10-fold concentration and finally diafiltration for 5 diavolumes against 20 mM sodium phosphate buffer, pH 7.0. Purification was carried out using Ni-charged chelating-sepharose as the primary capture, followed by a cation exchange step using an SP-sepharose column followed by a size exclusion column. The fractions containing the appropriate fusion proteins were concentrated using an SP-sepharose column. The purity and stability of the purified samples were assessed by SE-HPLC using a Varian ProSEC 300S column (Agilent Technologies, Santa Clara, CA, USA) and protein concentration estimated using the Micro BCA protein assay kit (Thermo Fisher Scientific, Rockford, IL, USA). For Western blot analysis, 20 ng of purified samples were loaded and immunoblotted as described previously whereas 2 μg were used for Coomassie staining.

### 4.6. Western Blot Analysis

Cell extracts were prepared in a lysis buffer (25 mM HEPES (pH 8.0), 150 mM NaCl, 10% glycerol, 0.5% sodium deoxycholate, 0.15% sodium dodecyl sulfate, 1 mM EDTA (pH 8.0), 3 mM MgCl_2_, 10 mM sodium fluoride, 1 mM dithiothreitol) supplemented with 0.6 μg/mL of aprotinin, leupeptin, bestatin, pepstatin, and phenylmethylsulfonyl fluoride, and 1 mM sodium orthovanadate. Antibodies for immunoblots include anti-Bcl-2 (5DE3; Cell Signaling, Danvers, MA, USA), anti-Bcl-xL (54H6; Cell Signaling), anti-Mcl-1 (D35A5; Cell Signaling), and anti-β-actin (Cell Signaling). All membranes were incubated with horseradish peroxidase-conjugated secondary antibodies (Cell Signaling) and visualized using enhanced chemiluminescence (Thermo Fisher Scientific, Rockford, IL, USA).

### 4.7. Cell Surface Reactivity

The cell surface reactivity of C6.5-diab and deB-C6.5-diab against HER2 positive SK-BR-3 tumor cells was determined by flow cytometry using a FACS Calibur (BD Biosciences, Mississauga, ON, Canada). Briefly, 2 × 10^5^ cells were incubated with the indicated amounts of C6.5-diab or deB-C6.5-diab for 2 h on ice. After washing with 2% FBS in PBS, bound C6.5 diabody and deB-C6.5-diab were detected with an anti-His antibody (GE Healthcare) at a 1/800 dilution followed by a fluorescein isothiocyanate (FITC) labeled goat anti-mouse antibody (Sigma) at a 1/200 dilution. Cells were analyzed on a FACS Calibur following propidium iodide (Molecular Probes, Eugene, OR, USA) staining and cell surface reactivity determined for live cells.

### 4.8. In Vitro Furin Cleavage Assay

Recombinant furin (2 units) (New England Biolabs Inc., Ipswich, MA, USA) was incubated with 5 µg of deB-C6.5-diab or C6.5-diab-deB in a buffer containing 100 mM Hepes (pH 7.5), 0.5% Triton X-100, 1 mM CaCl_2_ and 1 mM 2-mercaptoethanol. After 3 and 6 h at 25 °C, aliquots were removed and mixed with SDS-sample buffer to stop the reaction. Digested products and undigested control samples were separated by SDS-polyacrylamide gel electrophoresis and visualized by staining with Coomassie Brilliant Blue (Instantblue; Expedeon, San Diego, CA, USA).

### 4.9. Potency

Tumor cells were seeded at 5000 cells per well in a 96 well plate and allowed to adhere for 3 h at 37 °C. DeB-C6.5-diab, C6.5-diab-deB, T-deB, T-DM1, T-MMAE, or the corresponding free payloads were added to the cells over a range of concentrations and incubated for five days. Cell Titer 96 Aqueous One solution cell proliferation assay (Promega Corporation, Madison, WI, USA) or cell proliferation reagent WST-1 (Sigma) was used according to the manufacturer’s instruction to quantify relative cell viability. The IC_50_ value was interpolated from the resulting plot to determine the potency of the agents tested. 

To measure the viability of SK-BR-3, BT-474, and HCC1419 cells surviving a five-day treatment, 150,000 cells were seeded per well in a six-well plate and allowed to adhere for 3 h at 37 °C. Cells were treated with 10 nM deB-C6.5-diab, T-DM1, or T-MMAE. Cells surviving a five-day treatment and untreated cells were trypsinized and reseeded at 5000 cells per well in a 96-well plate. Viability was measured using Cell Titer 96 Aqueous One solution cell proliferation assay (Promega Corporation.) and reported as O.D._490_ values.

The potency of deB-C6.5-diab, T-DM1, and T-MMAE against BT-474-T-DM1, BT-474-T-MMAE, HCC1419-T-DM1, or HCC1419-T-MMAE cells was measured as follows. Briefly, 150,000 cells were seeded per well in a six-well plate and treated with 10 nM deB-C6.5-diab, T-DM1, or T-MMAE for five days. Treated cells were trypsinized and reseeded at a density of 5000 cells per well in a 96-well plate and allowed to adhere for 3 h at 37 °C. DeB-C6.5-diab, T-deB, T-DM1, T-MMAE, DM1, MMAE, taxol, and anti-EpCAM scFv-ETA were added over a range of concentrations and cells incubated for five days. Viability was reported relative to that of the non-treated control cells.

MDR was assessed using concomitant treatments with the inhibitors PSC833 (an MDR1 inhibitor), MK571 (an MRP1 inhibitor), and Ko143 (a BCRP inhibitor). The inhibitors were held at a fixed concentration in combination with 10 nM T-DM1 or T-MMAE. Doxorubicin (an MDR1 and MRP1 substrate) and irinotecan (a BCRP substrate) were used as controls. MDR inhibitors doxorubicin and irinotecan were purchased from Sigma. Viability was measured using Cell Titer 96 Aqueous One solution cell proliferation assay (Promega Corp.) and reported relative to that of the corresponding untreated control cells.

### 4.10. Tumorosphere Assays

Tumorosphere formation was assessed as previously described. Briefly, cells were trypsinized, placed in mammosphere media [DMEM/F12 (Life Technologies, Burlington, ON, Canada), 2% B27 supplement (Life Technologies, Burlington, ON, Canada), 20 ng/mL recombinant epidermal growth factor (Sigma), 0.5 µg/mL hydrocortisone (Stem Cell Technologies, Vancouver, BC, Canada), 5 µg/mL insulin (Sigma)] and resuspended as single cells using a 25-gauge needle [44]. Cells were plated in ultra-low-attachment six-well plates at a density of 10,000 cells/well and treated with deB-C6.5-diab, T-DM1, or T-MMAE at the time of plating. After 10 days, all tumorospheres greater than 50 µm in diameter were counted using an inverted microscope fitted with a graticule.

To assess the tumorosphere-forming efficiency of cells pretreated with deB-C6.5-diab, T-DM1, or T-MMAE, tumor cells were seeded at 150,000 cells per well in a six-well plate and allowed to adhere for 3 h at 37 °C. DeB-C6.5-diab, T-DM1, and T-MMAE were added to the cells at the indicated concentrations and incubated for five days. Cells were trypsinized and plated in mammosphere media as described above. The tumorosphere-forming efficiency was reported as a percentage relative to the corresponding untreated control.

### 4.11. Statistical Analysis

Differences were tested using the Student’s *t*-test; a *p*-value <0.05 was considered statistically significant.

## Figures and Tables

**Table 1:** Potency of deB-C6.5-diab, T-deB, T-DM1, and T-MMAE against carcinoma cell lines.

Cell Line	HER2 Expression	IC_50_ (nM)			
		deB-C6.5-diab	T-deB *	T-DM1 *	T-MMAE
**Breast**					
BT-474	3+	0.047 (0.005)	0.082 (0.019)	0.715 (0.025)	0.040 (0.002)
HCC1419	3+	0.155 (0.015)	0.086 (0.025)	1.900 (0.900)	5.705 (5.095)
HCC1569	3+	0.195 (0.025)	0.210 (0.080)	10.200 (0.200)	1.045 (0.155)
HCC1954	3+	0.043 (0.005)	0.045 (0.009)	0.320 (0.000)	0.076 (0.005)
HCC2218	3+	0.265 (0.065)	0.245 (0.095)	0.290 (0.042)	0.250 (0.050)
HCC202	3+	0.022 (0.010)	0.055 (0.015)	0.100 (0.049)	0.165 (0.055)
SK-BR-3	3+	0.330 (0.020)	0.275 (0.005)	0.047 (0.004)	0.037 (0.003)
MDA-MB-361	2+	0.685 (0.035)	1.795 (0.524)	0.320 (0.000)	0.051 (0.001)
MDA-MB-453	2+	0.225 (0.085)	0.335 (0.105)	0.440 (0.060)	0.255 (0.035)
MCF-7	1+	>10	>10	>10	>10
T47D	1+	>10	>10	8.000 (2.000)	>10
MDA-MB-231	0	>10	>10	>10	>10
**Lung**					
Calu-3	3+	0.041 (0.000)	0.105 (0.005)	1.400 (0.400)	0.086 (0.006)
**Gastric**					
NCI-N87	3+	0.032 (0.016)	0.09 (0.012)	0.265 (0.177)	0.091 (0.055)
OE-19	3+	0.043 (0.008)	0.050 (0.009)	0.037 (0.008)	0.0425 (0.007)

IC_50_ values derived from a minimum of two representative experiments with three replicates per dilution. Values in parentheses indicate standard error (S.E). * Non-original data as T-deB and T-DM1 IC_50_ values have previously been reported [30].

**Table 2:** Comparison of potency against untreated or T-DM1 or T-MMAE treated BT-474 or HCC1419 cells.

	HCC1419	HCC1419-T-DM1	HCC1419-T-MMAE	BT-474	BT-474-T-DM1	BT-474-T-MMAE
deB-C6.5-diab	0.15 (0.02)	0.19 (0.01)	0.17 (0.05)	0.07 (0.02)	0.33 (0.27)	0.11 (0.01)
T-deB	0.3 (0.05)	0.19 (0.038)	0.27 (0.03)	0.18 (0.005)	0.87 (0.02)	0.42 (0.17)
T-DM1	1.9 (0.9)	>10	>10	0.85 (0.25)	>10	>10
T-MMAE	5.7 (5.1)	>10	>10	0.04 (0.01)	>10	>10
T-Duo	0.28 (0.03)	4.65 (1.85)	>10	0.4 (0.1)	>10	9.95 (5.05)
DM1	135 (65)	>100	>100	17 (8)	>100	>100
MMAE	7 (3)	>100	>100	0.57 (0.17)	>100	>100
Taxol	>1000	>1000	>1000	18 (3)	>1000	>1000
Duocarmycin	0.65 (0.05)	3.55 (0.45)	4.95 (0.05)	0.31 (0.08)	5.45 (1.45)	3.05 (0.45)

IC_50_ values expressed in nM are the mean of a minimum of two representative experiments with three replicates per dilution. Values in parentheses indicate the S.E.

**Table 3:** Effect of deB-C6.5-diab, T-DM1, and T-MMAE on tumorosphere forming efficiency.

	BT-474	BT-474-T-DM1	BT-474-T-MMAE	HCC1419	HCC1419-T-DM1	HCC1419-T-MMAE
deB-C6.5-diab	0	0.14 (0.14)	0.6 (0.6)	2.9 (2.9)	0.19 (0.01)	1.8 (0.5)
T-DM1	91.7	42.8 (29.4)	72.6 (6.9)	91.3 (11.7)	110.6 (2.1)	83.6 (10.1)
T-MMAE	87.8	60.1 (28)	70.6 (6.1)	90.4 (13.6)	114 (6.1)	90.9 (12.9)

Tumorosphere forming efficiency values are expressed as % relative to the corresponding non-treated control and are the mean of a minimum of two independent experiments. Values in parentheses indicate the S.E.
